# A multimodal stacked ensemble model for cardiac output prediction utilizing cardiorespiratory interactions during general anesthesia

**DOI:** 10.1038/s41598-024-57971-6

**Published:** 2024-03-29

**Authors:** Albion Dervishi

**Affiliations:** Anaesthesiology and Intensive Care Medicine, Medius CLINIC NÜRTINGEN-Academic Teaching Hospital of the University of Tübingen, Auf dem Säer 1, 72622 Nürtingen, Germany

**Keywords:** Biotechnology, Computational biology and bioinformatics, Physiology, Cardiology, Medical research

## Abstract

This study examined the possibility of estimating cardiac output (CO) using a multimodal stacking model that utilizes cardiopulmonary interactions during general anesthesia and outlined a retrospective application of machine learning regression model to a pre-collected dataset. The data of 469 adult patients (obtained from VitalDB) with normal pulmonary function tests who underwent general anesthesia were analyzed. The hemodynamic data in this study included non-invasive blood pressure, plethysmographic heart rate, and SpO_2_. CO was recorded using Vigileo and EV1000 (pulse contour technique devices). Respiratory data included mechanical ventilation parameters and end-tidal CO_2_ levels. A generalized linear regression model was used as the metalearner for the multimodal stacking ensemble method. Random forest, generalized linear regression, gradient boosting machine, and XGBoost were used as base learners. A Bland–Altman plot revealed that the multimodal stacked ensemble model for CO prediction from 327 patients had a bias of − 0.001 L/min and − 0.271% when calculating the percentage of difference using the EV1000 device. Agreement of model CO prediction and measured Vigileo CO in 142 patients reported a bias of − 0.01 and − 0.333%. Overall, this model predicts CO compared to data obtained by the pulse contour technique CO monitors with good agreement.

## Introduction

Mechanical ventilation (MV) has a predictable impact on circulation^1,2^. Cardiorespiratory interactions are clinically important because MV can lead to cardiac instability^3^. MV typically uses positive airway pressure, thereby increasing intrathoracic pressure (ITP), causing a reduction in venous return, and increasing pulmonary vascular resistance, which can decrease preload and subsequently reduce cardiac output (CO). However, increased ITP can cause a decrease in the afterload on the heart, leading to increased stroke volume and CO.

In addition, excessive tidal volume and lung hyperinflation caused by overstimulation of sensory nerve endings located within the alveolar walls can lead to reflex bradycardia and depression of the somatic nervous system^4^.

Variations in arterial pulse and systolic pressure in mechanically ventilated patients with adjusted tidal volumes can predict fluid responsiveness during acute circulatory failure related to sepsis^5^. A decrease in CO_2_ concentration at the end-tidal concentration (EtCO_2_) in humans and animals correlates with a reduction in pulmonary blood flow/CO^6,7^. This relationship is significant and is currently implemented in anesthesia monitoring for non-invasive and minimally invasive breath-by-breath CO monitoring in patients ventilated during anesthesia and critical care^8^.

Presently, various methods for monitoring cardiovascular systems are available, including non-invasive, minimally invasive, and invasive techniques for measuring CO. Among these, thermodilution (TD) is considered the gold standard method, and pulse contour analysis is widely used^9,10^. A non-calibrated pulse pressure analysis device has been demonstrated to be clinically and statistically acceptable under hypo- and normodynamic conditions^11^.

CO is one of the most challenging hemodynamic parameters to assess in unstable patients. Even with a calibrated pulse contour hemodynamic monitoring system (VolumeView/EV1000), considerable overestimation of hemodynamic parameters has been reported when using a peripherally inserted central catheter from the brachial vein during calibration with temperature variation (ΔT) in comparison with a centrally inserted venous catheter^12^.

Moreover, more than a dozen non-invasive methods have been proposed and developed to estimate CO. The simplest of these methods involves calculating CO by multiplying the stroke volume (SV) by the heart rate (HR), where SV is obtained by multiplying the pulse pressure (systolic blood pressure (SBP)–diastolic blood pressure (DBP)) by a constant value (k = 2). This method has been evaluated and observed to have a moderate correlation between the measured and estimated CO (r = 0.60, p < 0.001)^13^. Furthermore, machine learning algorithms have been employed in animal models to predict CO accurately by utilizing waveform arterial blood pressure and HR, with a difference of − 0.13 (0.69 L/min) between the sheep’s pulmonary arterial blood flow using a transit time Doppler flow probe and predicted CO^14^.

Cardiorespiratory interaction data comprise heterogeneous information from the patient monitor, anesthesia machine, and CO monitor. Because of this diversity, employing a single model is impractical for comprehensively learning all facets of the data. Therefore, the rationale behind the use of multimodal stacking ensembles stems from their success in integrating multiple information sources for complex decision making in various medical machine learning tasks^15,16^.

Recently, there has been growing interest in applying machine learning algorithms to estimate CO, particularly from arterial pressure waveforms^14,17,18^. However, classical lumped parameter models, such as the Windkessel and Liljestrand–Zender models, suggest that approximate CO can be derived from basic monitoring data^13,19^. Therefore, we chose to incorporate cardiorespiratory interactions into the machine-learning prediction of CO based on numerical data. To the best of our knowledge, this is the first study to use this approach.

## Results

**Figure 1:**
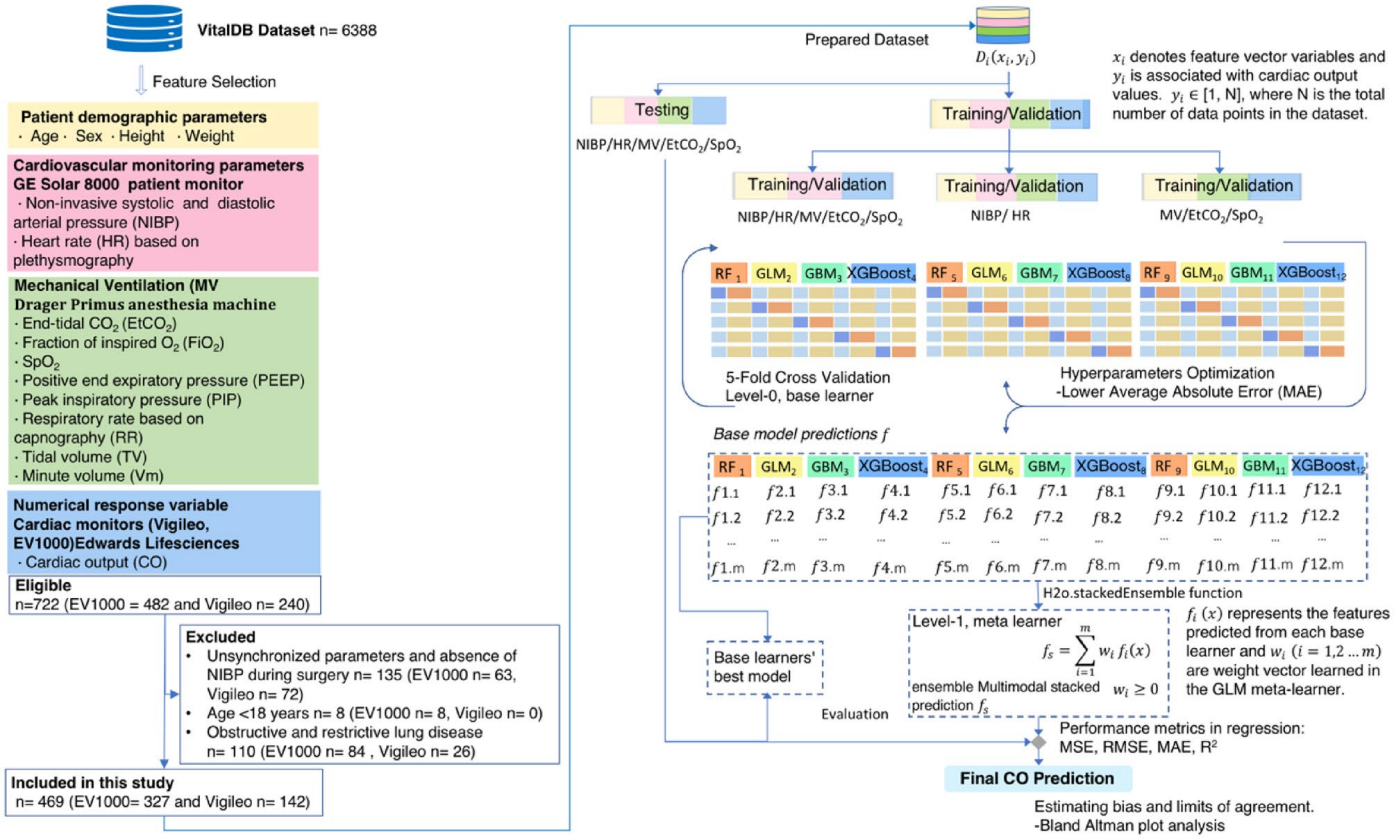
Data flow of multimodal stacking ensemble learning framework for cardiac output prediction during general anesthesia. Machine learning algorithms: Generalized linear model (GLM), random forest (RF), gradient boosting machine (GBM), and extreme gradient boosting (XGBoost).

Among the 6388 patients in the VitalDB Dataset, which measured hemodynamics including CO from the Vigileo and EV1000 devices and respiratory monitoring data, 722 were eligible for the study (Fig. 1). Patients < 18 years old were excluded (n = 8). Data were selected from the beginning to the end of surgery to ensure hands-free, automatic, and constant ventilation. Additionally, the absence of lung disease in patients undergoing general anesthesia could be achieved by selecting patients with normal pulmonary function testing from their clinical information. Consequently, exclusion criteria were applied to patients with abnormal pulmonary function tests (n = 110).

Participants with unsynchronized parameter measurements and an absence of intraoperative NIBP measurements were excluded (n = 135). After data preprocessing, 469 patients (EV1000, n = 327; Vigileo, n = 142) met the data requirements for further analysis. A summary of the patients and their clinical characteristics is presented in Supplementary Tables S1–3.

### Results of the base and multimodal stacked ensemble model regression

We evaluated the individual base learners and multimodal stacking ensemble regressions on three data subsets. We first trained and validated the hemodynamic response to cardiopulmonary interactions during MV using parameters from the hemodynamic and respiratory subsets (NIBP, HR, MV, SpO_2_, and EtCO_2_).

The hemodynamic data subsets (NIBP and HR) were then used for training and validation of the base models to predict CO from arterial blood pressure and heart rate.

Finally, respiratory data subsets (MV, EtCO_2_, and SpO_2_) were used for the training and validation of the base models and to calculate the hemodynamic effects of MV.

Table 1 presents the best results of the four base models, which were derived from the regression performance metrics of GLM, RF, GBM, XGBoost, and multimodal stacked ensemble models. For both CO monitoring devices, multimodal stacking outperformed all its base models in terms of the MSE, RMSE, and MAE, measuring 0.096, 0.31, and 0.186 for EV1000 and 0.057, 0.239, and 0.139 for Vigileo, respectively. In addition, based on MAE, average errors were evaluated. Compared to base models, RMSE was more sensitive to significant errors and multimodal stacking models predicted CO more accurately.

**Table 1:** Performance of the base and multimodal stacked ensemble models.

Best model	Data	MSE	RMSE	R2	MAE
Multimodal stacked ensemble	NIBP/HR/MV/SpO2/EtCO2/Target CO-EV1000	0.096	0.31	0.97	0.186
RF	NIBP/HR/MV/SpO2/EtCO2/Target CO-EV1000	0.12	0.346	0.968	0.197
XGBoost	NIBP/HR/MV/SpO2/EtCO2/Target CO-EV1000	0.123	0.35	0.966	0.211
XGBoost	NIBP/HR/ Target CO-EV1000	0.154	0.393	0.956	0.232
RF	NIBP/HR/ Target CO-EV1000	0.148	0.385	0.955	0.241
GBM	NIBP/HR/ Target CO-EV1000	0.166	0.408	0.953	0.256
GBM	NIBP/HR/ MV/SpO2/EtCO2/Target CO-EV1000	0.182	0.426	0.95	0.275
GBM	MV/SpO2/EtCO2/Target CO-EV1000	0.181	0.425	0.948	0.27
RF	MV/SpO2/EtCO2/Target CO-EV1000	0.192	0.438	0.948	0.262
XGBoost	MV/SpO2/EtCO2/Target CO-EV1000	0.251	0.501	0.927	0.343
GLM	NIBP/HR/MV/SpO2/EtCO2/Target CO-EV1000	1.81	1.345	0.444	1.064
GLM	NIBP/HR/ Target CO-EV1000	1.97	1.403	0.395	1.105
GLM	MV/SpO2/EtCO2/Target CO-EV1000	2.105	1.451	0.353	1.149
Multimodal stacked ensemble	NIBP/HR/MV/SpO2/EtCO2/Target CO-Vigileo	0.057	0.239	0.974	0.139
RF	NIBP/HR/MV/SpO2/EtCO2/Target CO-Vigileo	0.074	0.273	0.973	0.157
GBM	NIBP/HR/MV/SpO2/EtCO2/Target CO-Vigileo	0.078	0.28	0.972	0.164
XGBoost	NIBP/HR/MV/SpO2/EtCO2/Target CO-Vigileo	0.078	0.28	0.972	0.163
GBM	NIBP/HR/ Target CO-Vigileo	0.101	0.318	0.963	0.186
XGBoost	NIBP/HR/ Target CO-Vigileo	0.108	0.329	0.962	0.188
RF	NIBP/HR/ Target CO-Vigileo	0.1	0.316	0.959	0.198
RF	MV/SpO2/EtCO2/Target CO-Vigileo	0.115	0.34	0.956	0.2
GBM	MV/SpO2/EtCO2/Target CO-Vigileo	0.116	0.34	0.954	0.206
XGBoost	MV/SpO2/EtCO2/Target CO-Vigileo	0.132	0.363	0.951	0.219
GLM	NIBP/HR/MV/SpO2/EtCO2/Target CO-Vigileo	1.072	1.035	0.524	0.787
GLM	NIBP/HR/ Target CO-Vigileo	1.14	1.068	0.494	0.826
GLM	MV/SpO2/EtCO2/Target CO-Vigileo	1.599	1.264	0.291	0.981

Target CO measurements from the EV1000 and Vigileo monitoring devices. Machine learning algorithms: Generalized linear model (GLM), Random forest (RF), gradient boosting machine (GBM), and extreme gradient boosting (XGBoost).

*NIBP* non-invasive blood pressure, *HR* heart rate, *MV* mechanical ventilation, *SpO*_*2*_ oxygen saturation, *EtCO*_*2*_ end-tidal CO_2_.

### Multimodal stacked ensemble model CO prediction vs. measured CO from the arterial waveform analysis device

**Figure 2:**
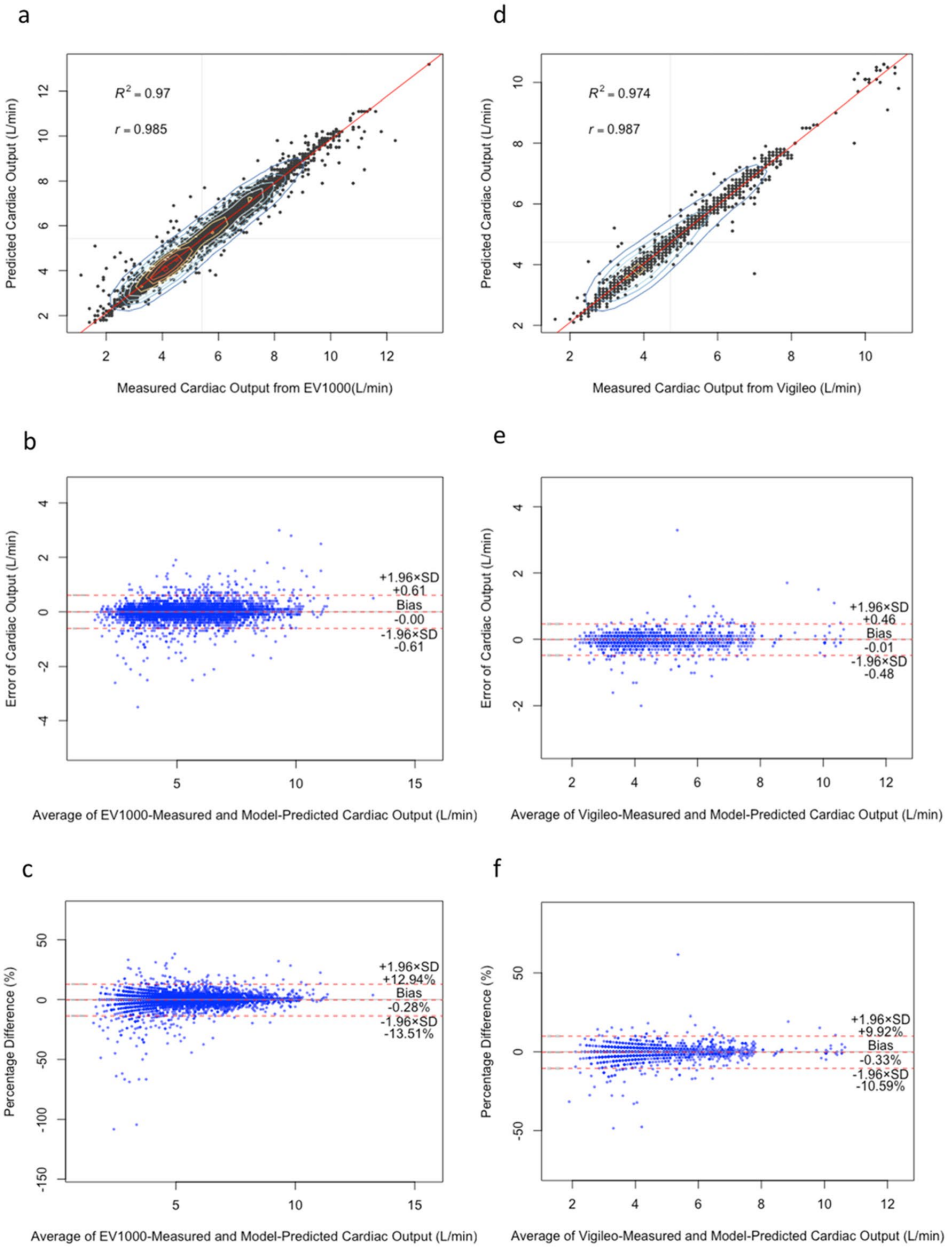
(**a**) and (**d**) show a scatterplot with spatial kernel density estimation^20^. In addition, they show a regression line between multimodal stacked ensemble model CO prediction and measured CO from EV1000 and Vigileo devices. (**b**), (**c**), (**e**) and (**f**) show descriptive statistics for the Bland–Altman analysis of agreement between model CO prediction and measured CO from the device.

Figure 2 and Table 2 present the baseline agreement between the multimodal stacked ensemble model for CO prediction and the CO measured using the EV1000 device. The difference between the methods was r = 0.985, and R^2^ was 0.97 (Fig. 2a). Bland–Altman analysis revealed that the mean difference between measurements and prediction was − 0.001 L/min (± 1.96 SD, 0.611, and − 0.614 L/min; Fig. 2b). The proportional mean difference was − 0.271% (± 1.96 SD, 12.94%, and − 13.488%; Fig. 2c).

**Table 2:** Bland–Altman analysis.

	Bland–Altman analysis	Mean value	CI at 95%	
			Lower CI	Upper CI
Agreement between multimodal stacked ensemble model CO prediction and measured EV1000 CO	Mean differences (Bias) (L/min)	− 0.001	− 0.01	0.007
	SD of Bias(L/min)	0.312		
	ULoA (+ 1.96 SD) (L/min)	0.611	0.595	0.626
	LLoA (-1.96 SD) (L/min)	− 0.614	− 0.629	− 0.598
	Percent differences (Bias) (%)	− 0.271	− 0.466	− 0.076
	SD of Bias (%)	6.743		
	ULoA (+ 1.96 SD) (%)	12.94	12.608	13.284
	LLoA (-1.96 SD) (%)	− 13.488	− 13.826	− 13.15
Agreement between multimodal stacked ensemble model co prediction and measured vigileo CO	Mean differences (Bias) (L/min)	− 0.01	− 0.018	0.005
	SD of Bias(L/min)	0.24		
	ULoA (+ 1.96 SD) (L/min)	0.464	0.443	0.485
	LLoA (-1.96 SD) (L/min)	− 0.477	− 0.497	− 0.456
	Percent differences (Bias) (%)	− 0.333	− 0.810	− 0.188
	SD of Bias (%)	5.233		
	ULoA (+ 1.96 SD) (%)	9.924	9.474	10.373
	LLoA (-1.96 SD) (%)	− 10.59	− 11.04	− 10.141

Lower and upper limits of agreement (LLoA and ULoA) and standard deviation (SD).

The agreement between the multimodal stacked ensemble model prediction of CO and the CO measured using Vigileo was r = 0.987, and R^2^ was 0.974 (Fig. 2d). The overall mean bias for agreement in CO was − 0.01 (± 1.96 SD, 0.464, and − 0.477 L/min; Fig. 2e). The proportional mean difference was − 0.333% (± 1.96 SD, 9.924%, and − 10.59%; Fig. 2f).

## Discussion

In this study, we proposed a multimodal stacking ensemble that combines data from non-invasive cardiovascular monitoring and MV parameters, including SpO_2_ and EtCO_2_. A fundamental principle of the proposed model is that stacking makes the prediction accuracy better than that of a single machine learning algorithm, and stacking several algorithms significantly improves the prediction accuracy. We demonstrate that the multimodal stacked ensemble model predicts accurate and valid CO values with marginal bias and a narrow CO limit of agreement compared with those obtained using pulse contour technique devices.

Ensemble stacking regression leverages multimodal information gathered from anesthesia machine and patient monitors, deriving benefits from the RF, GBM, and XGBoost base models. It effectively captures the nonlinear relationships in the interplay between the heart and lungs during positive-pressure ventilation. Non-linear interactions of cardiopulmonary features may explain why GLM base models exhibit inferior performance compared with other base models in hemodynamic and respiratory data. An additional advantage of ensemble stacking regression is the interpretability of the final predictions obtained using the GLM metalearner. Furthermore, it demonstrates robustness by harnessing the strengths of the multiple base models.

The Bland–Altman plot is widely recognized as the standard statistical method for assessing the agreement between two consecutive measurements of the same clinical variable^21^. When using a clinical CO measurement device, Bland–Altman plots do not indicate whether the LoAs are acceptable^22^. For example, an agreement limitation of ± 1 L/min may not be acceptable in patients with low CO syndrome. Additionally, our results include percentage difference plots demonstrating that multimodal stacked ensemble models accurately predict CO, with predictions falling within the acceptable clinical criteria (± 30%) of the proportional mean difference when compared those obtained using the Vigileo and EV1000 devices.

In previous studies the calibrated pulse wave analysis device EV1000 has proven to be accurate and consistent, and was thus used for our reference CO measurement. The results showed good agreement and interchangeability with TD CO measurement, with a bias of − 0.07 L/min, LoA of 2.0 L/min, and a percentage of 29%^23^. In addition, the uncalibrated FloTrac/Vigileo provides clinically acceptable accuracy under stable hemodynamic conditions, with an average error below 30% for CO compared with that obtained via TD^11^. However, severe sepsis and septic shock uncalibrated FloTrac/Vigileo vs. TD revealed no clinically acceptable tracking capability with a bias of − 0.86 L/min, LoA of − 4.48 to 2.77 L/min, and a percentage error of 48%^24^.

Our study was based entirely on non-cardiac surgery. Accordingly, NIBP was selected because it is a standard measurement for patients with ASA I and II and for intermediate-risk surgery. In addition, NIBP appears to be in acceptable agreement with invasively measured BP in patients with cardiogenic shock^25^, MV, and arrhythmia^26^. However, NIBP is not always well calibrated with invasive BP measurement, particularly in hypothermia and pronounced hypotension^25^. Although invasive BP, known as beat-by-beat measurement, is considered the gold standard method of diagnosis, NIBP is associated with fewer complications, particularly catheter-associated artery pseudoaneurysms, occlusions, and infections^27^. Occasionally, a measurement can be inaccurate owing to kinking or damping of the arterial line.

The HR was extracted from finger photoplethysmography and may represent acceptable accuracy based on electrocardiography (ECG) during normal breathing. Photoplethysmography and ECG-derived heart rates can differ moderately, and photoplethysmography shows an advantage in monitoring changes in ITP caused by ventilation, sleep apnea, and even changes in respiratory rate during deep breathing^28,29^.

Using the respiratory rate based on capnography, the expiratory tidal volume, and the expiratory Vm enabled us to obtain the exact delivered volume per breathing cycle recorded in the anesthesia machine (Fig. 6a–c). Noteworthy differences between the set and delivered tidal volumes have been demonstrated in several clinical situations, such as patient lung size, lung compliance, airway resistance, and maintenance of spontaneous breathing during general anesthesia through invasively assisted spontaneous ventilation^30,31^.

### Visualizing cardiopulmonary interactions and variable importance in a multimodal stacked ensemble model

Providing decision support using a functional hemodynamic machine learning model based on the complex relationship between the heart and lungs during general anesthesia should be understood by the medical environment. The predictability of the model was quantified in our work using partial dependence plots (PDPs)^32^, model parameter importance, and interaction variables^33^.

**Figure 3:**
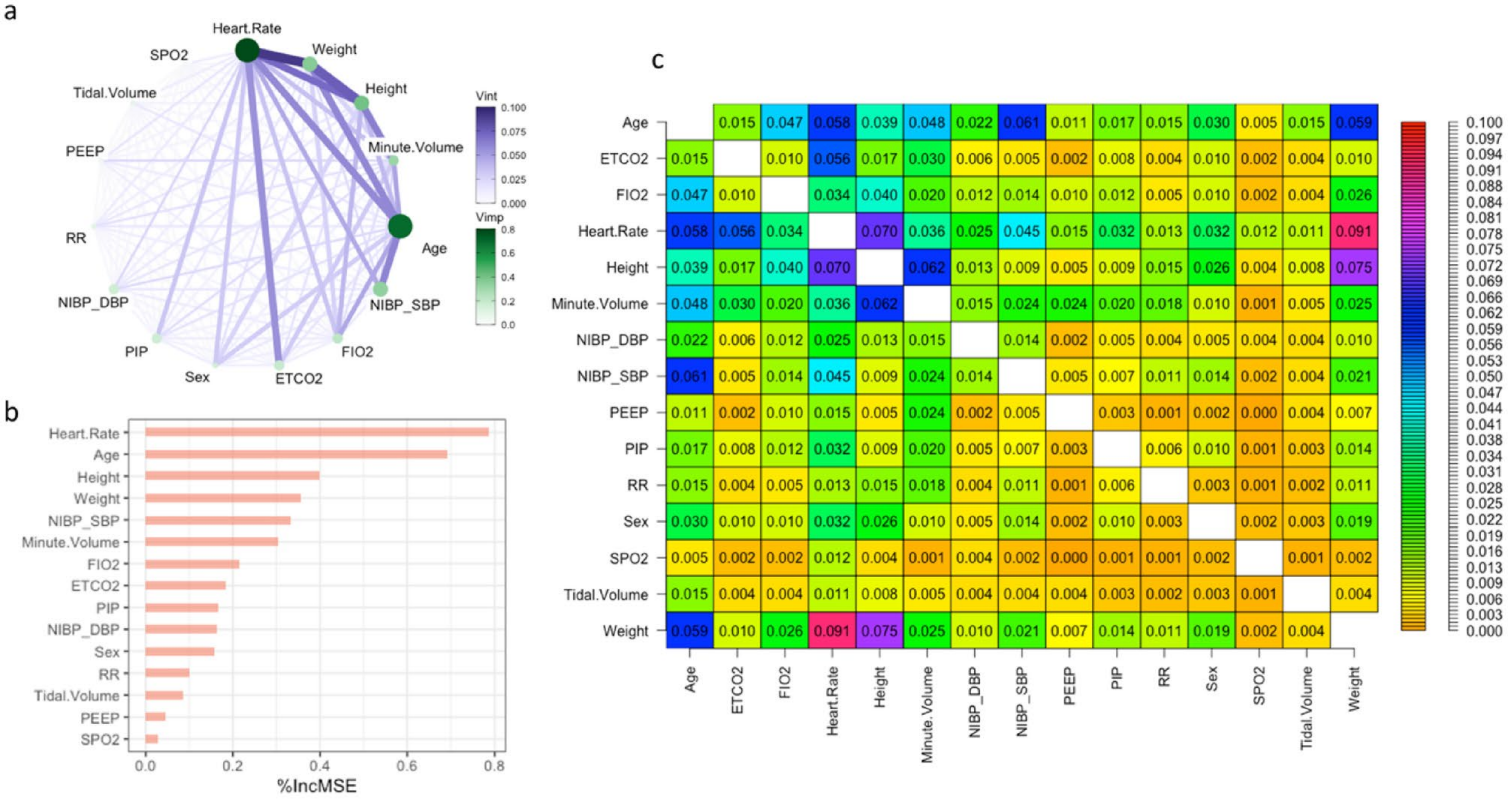
(**a**) The two-way interaction (Vint) represents the unnormalized Friedman's H-statistic between variables, depicted by connecting lines in the RF model for predicting the CO. The stronger the interaction, the thicker and darker the indigo line. The node's size and green intensity indicate the variable's importance (Vimp). (**b**) Contributions of explanatory variables to the RF model, measured in mean squared error "%IncMSE". (**c**) The table matrix presents the numerical values of the unnormalized Friedman’s H-statistic, indicating the interacting variables within the RF model for predicting the CO.

The symmetric matrix, derived from the calculation of variable importance and interactions using the RF model, was utilized to visualize the interaction variables in Table Fig. 3c, importance variables in Fig. 3b, and to construct a network graph in Fig. 3a. Variable importance is assessed exclusively based on changes in MSE. In difference, variable interactions are evaluated using the square root of the mean unnormalized version of the H-statistic, yielding a value on a scale of 0 to 1. This approach reduces the identification of spurious interactions and presents results by quantifying changes in the RMSE, which are measured on the same scale as CO in L/min. An RF model incorporating NIBP/HR/MV/SpO2/EtCO2 and CO measured by the Vigileo device was chosen to visualize the interaction and importance variables because it displayed the highest performance, with an R2 of 0.973 and an MSE of 0.074 compared to other base models.

All demographic, hemodynamic, and respiratory parameters displayed interactions to varying degrees with a range of H-statistic values (Fig. 3a and c). Hence, these plots facilitate the interpretation of cardiopulmonary interactions, particularly concerning total interactions and interactions between pairs of features, where one feature remains constant while others change, thereby influencing the accuracy of the cardiac output prediction. Demographic and hemodynamic variables, specifically weight and HR, were identified as the most important interactions, exhibiting an H-statistic value of 0.091. This finding suggests that an increase in the accuracy of the CO prediction corresponds to a reduction in the RMSE of 0.091 L/min. The constant pairs variable, HR, demonstrated the strongest reciprocal interactions with age (H-statistic = 0.058), NIBP-SBP (H-statistic = 0.045), height (H-statistic = 0.07), and EtCO2 (H-statistic = 0.056). The six variables that contributed the most to the prediction of CO in the RF model were HR, age, height, weight, NIBP-SBP, and minute volume (Fig. 3a and b).

**Figure 4:**
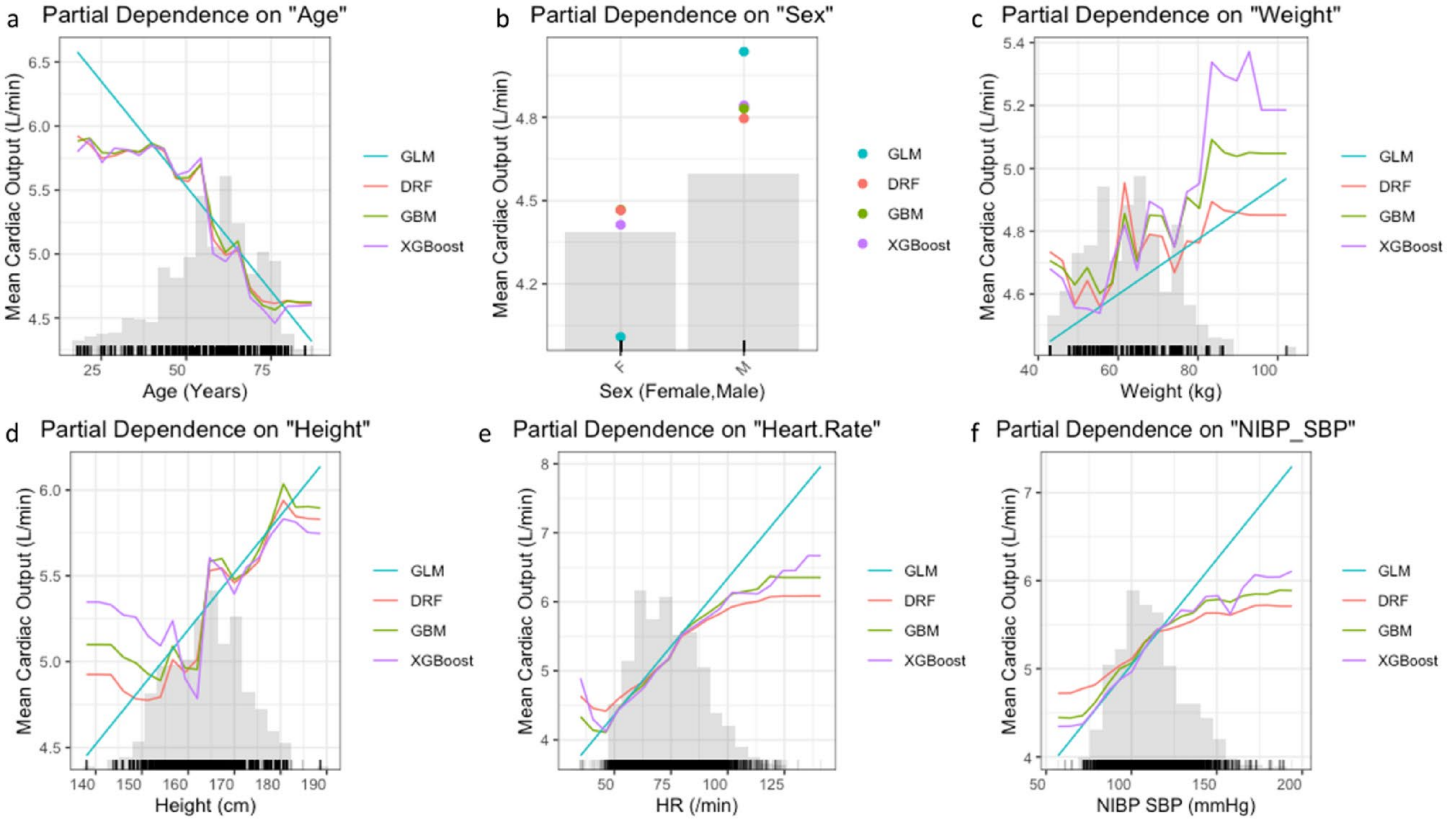
Partial dependence plots for variables in the multimodal stacking ensemble model for CO measured by Vigileo monitoring device. Partial Dependence Multimodel Plot gives a graphical depiction of the distributed random forest (DRF), gradient boosting machine (GBM), generalized linear model (GLM), and extreme gradient boosting (XGBoost). The effect of a variable is measured as the change in the mean cardiac output. *HR* plethysmographic heart rate, *NIBP SBP* systolic non-invasive blood pressure.

The results of our study were consistent with well-established data demonstrating that CO levels decrease with age by approximately 1% per year after the third decade (Fig. 4a). Age-related decline in the stroke index is accompanied by decreased body size and HR, which reduces CO^34^. We found the exact relationship between body size and CO in a straight-line regression, as observed in the last century^35^ (Fig. 4c,d). According to our findings, in females, one-way PDPs from the RF, GBM, and XGBoost models showed a decrease in CO of approximately 10% compared to those in males during intraoperative measurements. However, this difference was smaller than the 22% difference reported during the resting state^36^ (Fig. 4b).

HR is crucial to determining the diastolic filling time, influencing the SV via the Frank–Starling mechanism. For cardiopulmonary interactions during MV, venous return can be reduced, which can further compromise diastolic filling, particularly at high heart rates. Our study revealed a linear relationship between CO and HR up to 90/min, where deceleration began (Fig. 4e). Early curve deceleration is well documented in impaired right heart filling^37^. However, here, this may have been influenced by factors, such as autonomic nervous system activity, blood volume, and heart contractility, which were beyond the scope of this study.

**Figure 5:**
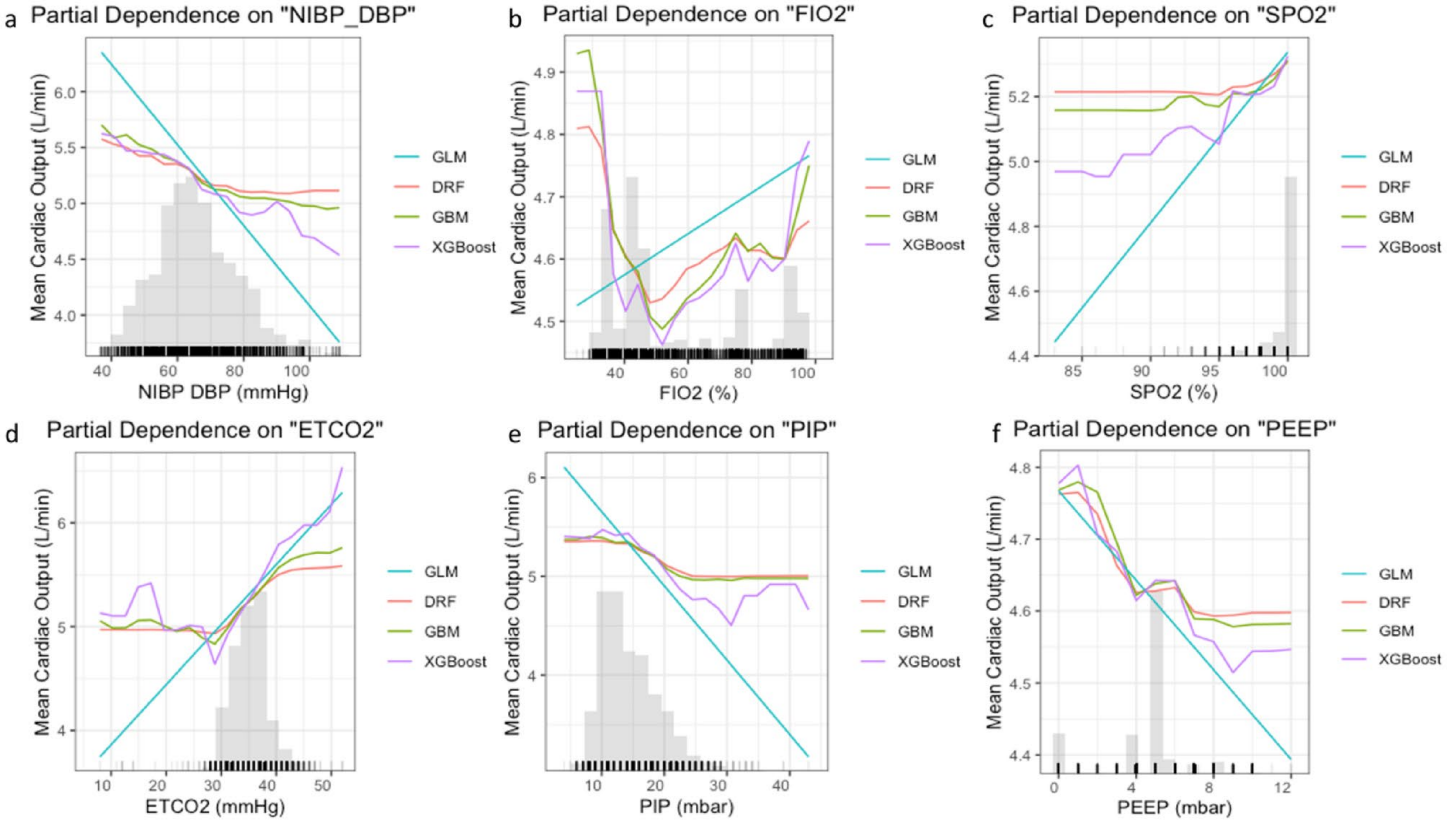
Partial dependence plots for variables in the multimodal stacking ensemble model for CO measured by Vigileo monitoring device. Partial dependence multimodel plot gives a graphical depiction of the distributed random forest (DRF), gradient boosting machine (GBM), generalized linear model (GLM), and extreme gradient boosting (XGBoost). The effect of a variable is measured as the change in the mean cardiac output. *NIBP*-*DBP* diastolic non-invasive blood pressure, *FiO2* fraction of inspired oxygen, *SPO2* oxygen saturation, *EtCO2* infrared spectrometry capnography, which measures end-tidal CO2, *PIP* peak inspiratory pressure, *PEEP* positive end-expiratory pressure.

The relationships between SBP, DBP, and CO during general anesthesia are complex and dynamic. In our study, we observed an increase in SBP corresponding to an increase in CO of up to 120 mmHg following the onset of the deceleration curve (Fig. 4f). The decreased CO level during high intraoperative SBP may be caused by increased vascular resistance, stiffened large arteries^38^, and reduced SV owing to elevated afterload. Our study demonstrated a decrease in DBP with a marginal increase in CO (Fig. 5a). An increase in pulse pressure might elucidate the observed increase in CO. An increase in SV owing to volume substitution results in increased CO, causing an increase in pulse pressure. Cardiopulmonary interactions and additional interventions such as vasopressor administration or adjustments to ventilator settings may play a substantial role. Additionally, the nonlinear relationship between pulse pressure, cardiac index (CI), and deceleration curve starting at a CI of 3 L/min/m^2^ has been well documented^39^.

One-way PDPs revealed an inverse relationship between CO and airway pressure (Fig. 5e). A decrease in SV and venous return is the primary mechanism by which increasing airway pressure reduces CO. The application of airway pressure levels at 10, 20, and 30 cm H_2_O led to a variation in the CI between + 6% and − 43%, which was associated with corresponding changes in the SV index (p < 0001, r2 = 0.89)^40^. Our findings align with those of earlier studies, as they indicated an increase in airway pressure during lung inflation and a reduction in CO at a rate of 0.5 L/min per 10 mbar increase in PIP.

PEEP increases ITP during the entire respiratory cycle to restore normal end-expiratory lung volume during MV. Increasing the PEEP levels allowed for greater lung expansion. PEEP during MV may also displace blood from the pulmonary circulation, increase mean systemic pressure, reduce venous return, and decrease CO and tissue perfusion^41^. Our model exhibits a decrease in the CO rate of 0.1 L/min by raising PEEP to 2.5 mbar (Fig. 5f). This decrease in CO with increasing PEEP in a curvilinear relationship has been previously reported^42^.

**Figure 6:**
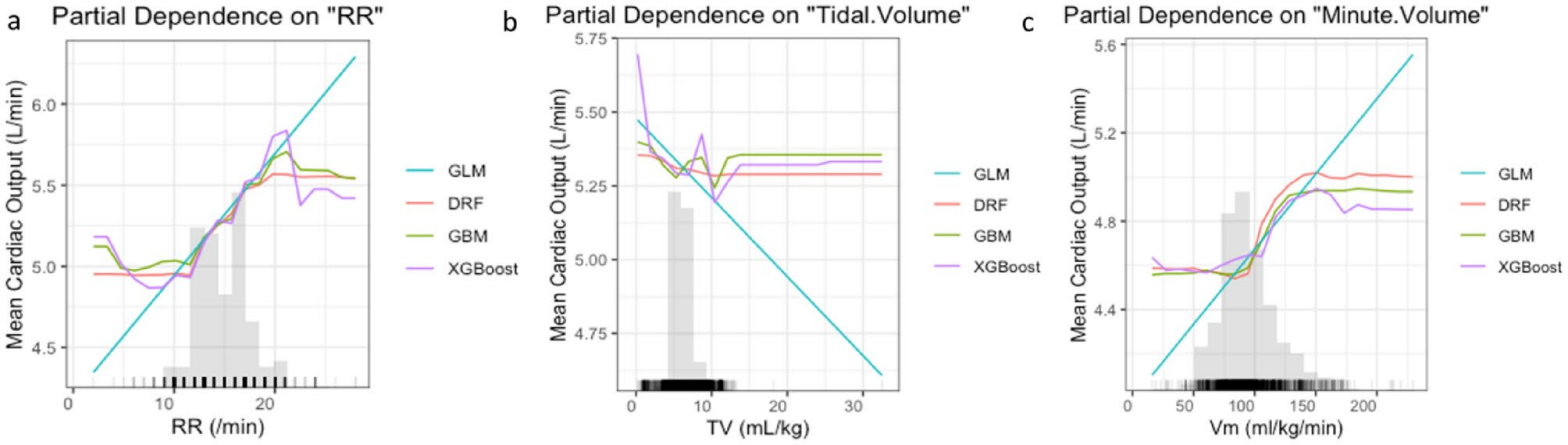
Partial dependence plots for variables in the multimodal stacking ensemble model for CO measured by Vigileo monitoring device. Partial dependence multimodel plot gives a graphical depiction of the distributed random forest (DRF), gradient boosting machine (GBM), generalized linear model (GLM), and extreme gradient boosting (XGBoost). The effect of a variable is measured as the change in the mean cardiac output. *RR* respiratory rate based on capnography, *TV* expiratory tidal volume, *Vm* expiratory minute volume.

A reduction in TV increases CO; nevertheless, the degree of improvement in hemodynamics depends largely on ITP^43^. Reducing the tidal volume increases chest wall compliance by decreasing ITP during MV and increasing venous return, leading to increased left ventricular preload and CO. This is consistent with our finding; our model showed an increase in CO of 0.03 L/min per 1 mL/kg of TV reduction (Fig. 6b). A tidal volume > 15 mL/kg markedly decreases HR and blood pressure and reduces CO^44^. However, we could not evaluate this observation with limited training data for tidal volumes > 15 mL/kg, and a machine learning model could not make meaningful predictions.

Changes in exhaled carbon dioxide during general anesthesia with stable ventilation correspond to changes in CO and metabolic CO_2_ production^45^. At ETCO_2_ levels > 30 mmHg, RF, GBM, and XGBoost models predict a satisfactory CO increase of 0.5 L/min per 10 mmHg of ETCO_2_ (Fig. 5d). A similar correlation between ETCO_2_ and CO has been reported in previous studies^46^. An animal model during cardiopulmonary resuscitation showed a correlation coefficient of 0.79 between EtCO_2_ and CI^47^. This finding is consistent with that of the GLM model. However, the GLM model had a lower performance than that of the RF, GBM, and XGBoost models and had less training data with EtCO_2_ < 30 mmHg.

A decline in SpO_2_ was observed with decreasing CO in all base models in our study (Fig. 5c). Decreased CO caused by cardiopulmonary interactions is the primary factor in the reduction of arterial oxygen content observed during MV^48^. Hypovolemia and vasodilation, which are commonly observed during general anesthesia, may also contribute to this phenomenon. However, our data did not allow us to determine whether the increased inspired O_2_ fraction reflected an increase in CO (Fig. 5b). It is widely recognized that increases in FiO_2_ at fixed values of CO fail to detect conditions of low oxygen supply during central venous oxygen saturation^49^.

This study may be more compelling if the model was applied to a dataset that included direct CO measurements obtained through thermodilution using a pulmonary artery catheter. Nevertheless, the interpretability of the developed multimodal stacking ensemble is a notable strength of the proposed system. By offering valuable insights into the interpretation of the model, we deepen our understanding of all purely physiological inputs implicated in CO estimation. This not only enhances scholarly comprehension within the discipline, but also promotes the endorsement and integration of the system among healthcare practitioners. The architecture of this model aligns with the characteristics of "locked" algorithms as defined in the proposed regulatory framework for modifications to Artificial intelligence/machine learning (AI/ML)-based Software as a Medical Device by the food and drug administration (FDA)^50^. Training the complex algorithm with numerical data enhanced its versatility, allowing the model to be saved, exported, and deployed in diverse medical environments for production use.

Further limitations of this study are as follows. The data analyzed was from one source only and focused solely on adult patients. During data mining, we could not find synchronized records of sudden blood loss or vasoactive infusions. This limitation has an impact on our model's ability to assess fluid responsiveness and requires thorough evaluation when our model undergoes testing in real-time general anesthesia scenarios. The perioperative clinical information dataset contained data on estimating intraoperative blood loss and cumulative intraoperative use of vasoactive medications (ephedrine, phenylephrine, and epinephrine). However, this information lacks a recorded time, making it unsuitable for inclusion in our model. Mechanical ventilation without spontaneous effort may affect hemodynamics differently; nonetheless, the ventilation modes were not documented in the VitalDB data, leading to their exclusion from this study. In addition, the small number of patients with obstructive or restrictive lung diseases made it difficult to include them in the data subset. Although constant ventilation was ensured during surgery in this study, it is important to recognize that the period from the onset of anesthesia to the start of surgery and the time between the end of surgery and extubation are important for comprehending the influence of cardiopulmonary interactions on hemodynamics. During extubation or weaning, spontaneous inspiratory efforts in patients with obstructive and restrictive lung disease may strongly decrease CO by increasing the left ventricular afterload, especially if left ventricular function is already impaired^43^. Our model should be improved in the future to address these cardiopulmonary interactions.

## Conclusion

Using a multimodal stacking ensemble algorithm, involving two-component regression based on hemodynamic and respiratory monitoring inputs, acceptable performance was achieved in comparison to data obtained by the pulse contour technique CO monitors.

This innovative methodology has the potential to discern the intrinsic physiological processes occurring in cardiopulmonary interaction during mechanical ventilation at a 14-s interval, particularly in the context of CO estimation. Based on the last recorded monitoring parameter, the model predicts only current CO for each interval of 14 s. By predicting CO cumulatively over time, we can assess the impact of cardiopulmonary interaction on CO during mechanical ventilation.

Current research has the potential to address the rising demand for non-invasive CO measurements; however, it is crucial to conduct external validation using several data sources and diverse patient conditions.

## Methods

### Data source

In this study, de-identified data were used from an open database of non-cardiac surgery patients who underwent routine or emergency operations at Seoul National University Hospital, Seoul, Korea, from August 2016 to June 2017^51^. This database contains prospectively collected intraoperative vital sign data from 6,388 general, thoracic, urological, and gynecological surgery cases, with the formal approval of an ethics committee/IRB (H-1408-101-605) and registered at www.clinicaltrials.gov (identifier: NCT02914444). Perioperative clinical information was retrospectively obtained. In addition, several anesthesia devices recorded up to 12 waveforms and 184 numeric data tracks during surgery using the Vital Recorder program.

### Monitoring parameters and data structure

Hemodynamic data, obtained as numeric values at 2-s intervals, included non-invasive blood pressure (NIBP), plethysmographic HR, and SpO_2_ data (Solar™ 8000 M, GE healthcare, Wauwatosa, WI, USA). In addition, CO was recorded using pulse contour technique monitors such as Vigileo and EV1000 (Edwards Lifesciences, Irvine, CA, USA).

Respiratory data collected using the anesthesia machine (Primus, Dräger, Lübeck, Germany) were recorded at 7-s intervals. MV was determined by estimating the fraction of inspired oxygen (FiO_2_), expiratory TV, expiratory minute volume (Vm), positive end-expiratory pressure (PEEP), peak inspiratory pressure (PIP), respiratory rate based on capnography, and infrared spectrometry capnography, which measures EtCO_2_, thereby ensuring adequate and accurate ventilation per period.

According to the official VitalDB data descriptor, invalid data tracks that were identified during the data check were excluded. Following this exclusion, the data were organized into nonconstant time intervals^51^.

Data extraction occurred at a rate of one second per interval, involving 16 parameters and comprising a total of 6,236,640 rows over a duration of 1732.4 h (EV1000:1288.03 h; Vigileo: 444.1 h) intraoperative monitoring with 99,786,240 datapoints. Hemodynamic monitoring data, collected at 2-s intervals, were aligned with the anesthesia machine data and recorded at 7-s intervals. Consequently, data synchronization occurred every 14 s, encompassing 538,354 rows of data. CO, marked as a target intraoperative parameter, demonstrated a 43.5% reduction in missed data, leaving 234,225 rows.

The NIBP measurements in VitalDB were recorded every 2 s. However, intraoperative NIBP in this data subset was intermittently measured over a period of 2–45 min, with interval data ranging from 1 to 10 min recorded for each measurement. The absence of NIBP during surgery led to a 77.9% reduction in data. After extracting data for adult patients aged > 18 (constituting 2.4% of the dataset) and removing the remaining 1% of missing values, a total of 49,007 pairs of row measurement data were available for subsequent analyses. To uphold the rigor of the analysis and enhance the precision of the results, rows containing missing values were excluded from the dataset.

### Retrieving demographic and patient characteristics

The VitalDB records were identified using case IDs that could be matched with the case IDs in the perioperative clinical information. After data preprocessing, each record was matched to its corresponding perioperative clinical information to retrieve the demographic and patient characteristics. Subject characteristics, including age, sex, weight, height, body mass index (BMI), ASA grade, preoperative comorbidity, department, operation type, surgical approach, and postoperative ICU stay, were analyzed. In addition to describing the parameters, descriptive statistics were used to describe them in terms of minimum, maximum, mean, standard deviation, median, 25th–75th quartiles, and 95% confidence intervals (see Supplementary Tables S1–3).

### Evaluating levels of general anesthesia

Both inhalational and intravenous anesthetics influence systemic vascular resistance and cardiac contractility, leading to a reduction in CO^52^. Therefore, it is imperative to delineate the depth of anesthesia while maintaining general anesthesia in this project. In instances of total intravenous anesthesia (TIVA), propofol was titrated to maintain the bispectral index (BIS) at 41.07 ± 9 and delivered through a target-controlled infusion pump. For inhalational anesthesia, sevoflurane and desflurane were adjusted using a vaporizer to target the Minimum Alveolar Concentration (MAC) at 0.87 ± 0.24 independently of the BIS value. The depth of anesthesia administered during general anesthesia for this study is delineated in Supplementary Table 5.

### Model development

For multisystem CO prediction, we used multimodal stacking-based ensemble learning regression techniques (Fig. 1). We split the multimodal data into training, validation, and test sets at a ratio of 7.5:1.25:1.25.

After preprocessing, the training and validation data were used to train and validate the model using both hemodynamic and respiratory parameters (NIBP, HR, MV, SpO_2_, and EtCO_2_), to calculate the relationship between cardiorespiratory interactions and CO. Further training and validation data were separated into hemodynamic (NIBP and HR subsets) and respiratory parameters (MV, SpO_2_, and EtCO_2_ subsets). Three data subsets were constructed using the demographic variables (age, height, weight, and sex).

Four base learner models were used for the multimodal stacking ensemble in this study (Level-0): a generalized linear model (GLM)^53^, Random Forest (RF)^54^, Gradient Boosting Machine (GBM)^55^, and extreme gradient boosting (XGBoost)^56^. Excluding GLM, all other models were nonlinear regressions.

Within the R interface for ‘H_2_O’, a scalable open-source platform, we employed a random grid search methodology to pinpoint an optimal set of hyperparameter values for maximizing the effectiveness of our models on the dataset. The H2O platform employs a random hyperparameter search with time and metric based early stopping, enabling the identification of high-quality models within a limited computational timeframe^32,57^.

To optimize the hyperparameters of the GLM, distributed RF, GBM, and XGBoost regression models in the three data subsets, a random search was conducted by splitting the training set fivefold to optimize the hyperparameters and enhance the model performance by lowering the predicted value error measured through the mean absolute error (MAE). The key hyperparameters for DRF, GB, and XGBoost (ntrees, max_depth, learn_rate, sample_rate/col_sample_rate, and min_rows) were employed, whereas for GLM, alpha and lambda were utilized. Additionally, search criteria including max_models, max_runtime_secs, stopping_tolerance, and stopping_rounds were applied. The hyperparameters of the base models are summarized in Supplementary Table 4.

The best optimized regression predictions of the 12 base models from the three data subsets were subsequently used as input features for the multimodal stacking ensemble method (Level-1)^15^.

In the second step, the Stacked Ensemble method uses a metalearning algorithm to learn the optimal combination of the base learners^58^. The metalearner is a default H2O GLM with non-negative weights. The GLM metalearner was evaluated during the implementation of a stacking regression with cross-validation, where lambda was employed as a hyperparameter.

The comparison results of the base models and multimodal stacked assembly with the performance metrics in the regression are listed in Table 1.

### Performance metrics in regression

The mean square error (MSE), root mean square error (RMSE), MAE, and coefficient of determination (R^2^) were used as performance indicators to evaluate the regression algorithms. The MSE and RMSE are commonly used regression metrics.

1 $$RMSE=\sqrt{\frac{1}{N}} \sum_{i = 1}^{N}{({y}_{i}-{y}_{i}^{p})}^{2}.$$

By taking the square root of MSE, the RMSE (Eq. 1) measures the difference between the predicted CO ${y}_{i}^{p}$ and measured CO values ${y}_{i}$. The RMSE was calculated as the square root of the sum of all regression errors per row divided by the total number of observations. The regression performance improved, with lower RMSE and MSE values.

2 $$MAE=\frac{1}{N}\sum_{i = 1}^{N}\left[{y}_{i}-{y}_{i}^{p}\right].$$

The MAE measures the difference between the measured CO ${y}_{i}$ and predicted CO ${y}_{i}^{p}$ values divided by the total number of observations (Eq. 2). Low MAEs indicate high model accuracy.

3 $${R}^{2}=1-\frac{\sum_{i=1}^{N}{\left({y}_{i}-{y}_{i}^{p}\right)}^{2}}{\sum_{i=1}^{N}{\left({y}_{i}-\overline{y}\right)}^{2}}.$$

R^2^ represents how well a regression model explains the variability between the measured and predicted CO. From 0 to 1, the higher the value, the better the model (Eq. 3). R^2^ represents the variability explained by the model (squared difference between the target ${y}_{i}$ and the predicted value ${y}_{i}^{p}$) divided by the total deviance y value (squared difference between the target ${y}_{i}$ and the mean target values $\overline{y}$).

### Model evaluation

We used the Bland–Altman method as a statistical standard to compare the measures of CO ${y}_{i}$ from both EV 1000 and Vigileo with the multimodal stacking ensemble model prediction CO ${y}_{i}^{p}$^59^. The Bland–Altman plot was introduced to describe the agreement, where the y-axis shows the difference between the measured and model-predicted CO (${y}_{i}-{y}_{i}^{p}$), and the x-axis represents the average of the measured and model-predicted CO ((${y}_{i}$+${y}_{i}^{p}$)/2). In summary, the absolute mean difference between the measured and predicted CO ($\overline{d}=\frac{1}{N}\sum_{i=1}^{N}{(y}_{i}-{y}_{i}^{p}$) can be used to estimate the constant bias, and the limits of agreement (LoAs) lie between $\overline{d}-$ 1.96S_d_ and $\overline{d}$+1.96S_d_, where S_d_ is the standard deviation. Percentage difference plots and analyses were used to assess the proportional differences between the measured and model-predicted CO. This shows how this error influences lower CO measurements, whereas for higher CO values, the percentage bias is decreased^60^. In the proportional Bland–Altman plots, bias changes over the measuring range, and are presented as a proportional mean difference $\overline{\overline{d}}$= $\frac{1}{N}\sum_{i = 1}^{N}\frac{({y}_{i} - {y}_{i}^{p})}{({y}_{i}+{y}_{i}^{p})/2}$, and the LoA lay between $\overline{\overline{d}}-$ 1.96S_d_ and $\overline{\overline{d}}$+1.96S_d_.

### Supplementary Information

Supplementary Tables S1–S3.

Supplementary Table S4.

Supplementary Table S5.

## Data Availability

The datasets generated and/or analysed during the current study are available in the VitalDB repository, https://physionet.org/content/vitaldb/1.0.0/. The R-based interactive web applications for this study can be accessed at the address provided below: https://albiondervishi.shinyapps.io/CO_Shiny/.
