# Supplementary material for: A multimodal stacked ensemble model for cardiac output prediction utilizing cardiorespiratory interactions during general anesthesia
Source: Sci Rep. 2024 Mar 29;14:7478. doi: 10.1038/s41598-024-57971-6 (PMC10980739; doi:10.1038/s41598-024-57971-6)
Supplement: Supplementary file 1 — Supplementary Tables S1–S3. [file 41598_2024_57971_MOESM1_ESM.docx]

|  | **EV1000 (n=327)** | | **Vigileo (n=142)** | | |
| --- | --- | --- | --- | --- | --- |
|  | Mean (SD) | Min-Max | | Mean (SD) | Min-Max |
| Age, years | ﻿﻿56.59±14.2 | 18-87 | | ﻿59.1±13.5 | ﻿18-89 |
| ﻿Sex n (%) |  |  | |  |  |
| Male | ﻿204(62.4) |  | | ﻿82(52.7) |  |
| Female | ﻿﻿123(37.6) |  | | ﻿60(42.3) |  |
| Height, cm | ﻿163.62±﻿(8.3) | ﻿138-188.6 | | ﻿163.2±(7.8) | ﻿145.4-﻿183.3 |
| Weight, kg | ﻿61.89±﻿(11.7) | 26.9-139.7 | | ﻿62.6±(10.1) | ﻿42.9-102.2 |
| Body mass index, kg/m2 | ﻿23.01±﻿(3.6) | 11.5-43.2 | | ﻿23.4±(2.8) | ﻿15.9-34.1 |
| ASA grade, n (%) | 320 |  | | 139 |  |
| 1 | ﻿55(17.2) |  | | ﻿35 (25.2) |  |
| 2 | ﻿193(60.3) |  | | ﻿﻿94(67.6) |  |
| 3 | ﻿69(21.6) |  | | ﻿10(7.2) |  |
| 4 | ﻿3(0.9) |  | | ﻿- |  |
| 5 | - |  | | ﻿- |  |
| Preoperative comorbidity, n (%) |  |  | |  |  |
| Hypertension | ﻿122(37.3) |  | | ﻿56 (39.4) |  |
| Diabetes mellitus | 43(13.1) |  | | ﻿16 (11.3) |  |
| Pulmonary function test, n (%) |  |  | |  |  |
| Normal | ﻿327(100) |  | | ﻿142 (100) |  |
| Departments |  |  | |  |  |
| ﻿General surgery | ﻿327(100) |  | | ﻿37(26.1) ﻿ |  |
| ﻿Gynecology | - |  | | ﻿3 (2.1) |  |
| ﻿Thoracic surgery | - |  | | ﻿83 (58.5) |  |
| ﻿Urology | - |  | | ﻿19 (13.4) |  |
| Operation type, n (%) | - |  | | - |  |
| ﻿Transplantation | 99(30.3) |  | | - |  |
| ﻿Hepatic | 92(28.1) |  | | ﻿2 (1.4) |  |
| Major lung resection | - |  | | ﻿46 (32.4) |  |
| ﻿Minor lung resection | - |  | | 37 (26.1) |  |
| ﻿Biliary/Pancreas | 52(15.9) |  | | 5(3.5) |  |
| ﻿Stomach | 10(3.1) |  | | ﻿7 (4.9) |  |
| ﻿Colorectal | 17(5.2) |  | | ﻿7 (4.9) |  |
| ﻿Breast | 3 (0.9) |  | | - |  |
| ﻿Thyroid | 3(0.9) |  | | ﻿8 (5.6) |  |
| Vascular | 12(3.7) |  | | ﻿4(2.8) |  |
| ﻿Others | 39(11.9) |  | | ﻿26 (18.3) |  |
| Approach, n (%) |  |  | |  |  |
| Open | ﻿239(73.1) |  | | ﻿25 (17.6) |  |
| Videoscopic | ﻿﻿﻿88 (26.9) |  | | ﻿68 (47.9) |  |
| Robotic | - |  | | ﻿49 (34.5) |  |
| Postop ICU stay, day (SD) | ﻿0.79±1.4 | 0-8 | | 0.5±1.1 | 0-8 |

**Table S1.** Data characteristics

|  | Min | Max | Median | Q1 | Q3 | IQR | Mad | Mean | SD | SE | CI (95%) |
| --- | --- | --- | --- | --- | --- | --- | --- | --- | --- | --- | --- |
| Age (Years) | 18 | 87 | 58 | 49 | 66 | 17 | 13.3 | 56.5 | 14.0 | 0.065 | 0.128 |
| Height(cm) | 138 | 188.6 | 164.5 | 158.1 | 169.9 | 11.8 | 8.8 | 164 | 8.2 | 0.038 | 0.075 |
| Weight(kg) | 26.9 | 139.7 | 60.7 | 54.1 | 68.2 | 14.1 | 10.6 | 61.9 | 11.4 | 0.053 | 0.104 |
| ETCO2(mmHg) | 5 | 53 | 35 | 33 | 37 | 4 | 2.9 | 35.1 | 3.2 | 0.015 | 0.03 |
| FIO2(%) | 23 | 100 | 36 | 35 | 40 | 5 | 2.9 | 39.7 | 11.2 | 0.053 | 0.103 |
| PEEP (mbar) | 0 | 10 | 0 | 0 | 0 | 0 | 0 | 0.6 | 1.7 | 0.008 | 0.016 |
| PIP (mbar) | 4 | 53 | 14 | 11 | 17 | 6 | 4.4 | 14.5 | 4.4 | 0.021 | 0.04 |
| RR(/min) | 1 | 37 | 15 | 14 | 17 | 3 | 1.4 | 15.1 | 2.3 | 0.013 | 0.025 |
| TV (ml(kg) | 0.2 | 32.5 | 5.9 | 5.4 | 6.6 | 1.2 | 0.9 | 6.1 | 1.1 | 0.005 | 0.01 |
| Vm(ml/kg/min) | 6.9 | 336.5 | 89.2 | 78.3 | 100.5 | 22.2 | 16.1 | 90.8 | 18.5 | 0.087 | 0.17 |
| NIBP-SBP (mmHg) | 57 | 199 | 109 | 97 | 123 | 26 | 19.2 | 111.3 | 20.5 | 0.096 | 0.188 |
| NIBP-DBP (mmHg) | 32 | 109 | 65 | 57 | 74 | 17 | 11.8 | 65.7 | 12.6 | 0.059 | 0.116 |
| HR (/min) | 34 | 141 | 73 | 62 | 84 | 22 | 16.3 | 73.9 | 15 | 0.07 | 0.138 |
| SPO2(%) | 83 | 100 | 100 | 100 | 100 | 0 | 0 | 99.5 | 1. | 0.005 | 0.01 |
| EV1000 CO(L/min) | 1.1 | 15.5 | 5 | 3.9 | 6.6 | 2.7 | 1.9 | 5.3 | 1.7 | 0.008 | 0.016 |

**Table S2.** Numeric data are presented as minimum, maximum, Q-Quartile, Median, Mean, SD-Standard deviation, IQR-interquartile range, Mad-mean absolute deviation, SE-standard error, and CI-confidence interval, measured 1288.03 hours of intraoperative monitoring data from 327 patients. EV1000 CO-Cardiac output measured using the EV1000 device. NIBP-SBP and NIBP-DBP- Systolic and Diastolic noninvasive blood pressure, HR-plethysmographic heart rate, FiO2-Fraction of inspired oxygen, TV- Expiratory tidal volume, Vm-expiratory minute volume, RR-respiratory rate, PEEP-positive end-expiratory pressure, PIP-peak inspiratory pressure, EtCO2- infrared spectrometry capnography, which measures end-tidal CO2.

|  | Min | Max | Median | Q1 | Q3 | IQR | Mad | Mean | SD | SE | CI (95%) |
| --- | --- | --- | --- | --- | --- | --- | --- | --- | --- | --- | --- |
| Age (Years) | 18 | 89 | 61 | 56 | 70 | 14 | 10.3 | 59.3 | 13.5 | 0.105 | 0.206 |
| Height(cm) | 145.4 | 183.3 | 162.8 | 157.3 | 170 | 12.7 | 8.8 | 163.2 | 7.9 | 0.062 | 0.121 |
| Weight(kg) | 42.9 | 102.2 | 63.4 | 54.8 | 68.9 | 14.1 | 11.4 | 62.8 | 10.6 | 0.083 | 0.162 |
| ETCO2(mmHg) | 5 | 57 | 35 | 32 | 37 | 5 | 2.9 | 35.1 | 3.9 | 0.031 | 0.06 |
| FIO2(%) | 22 | 98 | 45 | 41 | 76 | 35 | 16.3 | 56.6 | 21.9 | 0.171 | 0.334 |
| PEEP (mbar) | 0 | 12 | 5 | 4 | 5 | 1 | 0 | 4.4 | 1.9 | 0.015 | 0.03 |
| PIP (mbar) | 4 | 51 | 19 | 16 | 23 | 7 | 4.4 | 19.1 | 5.4 | 0.042 | 0.083 |
| RR(/min) | 1 | 33 | 15 | 13 | 17 | 4 | 2.9 | 14.9 | 2 | 0.01 | 0.043 |
| TV (ml/kg) | 0.2 | 26.2 | 6.4 | 5.5 | 7.6 | 2.1 | 1.5 | 6.6 | 1.6 | 0.012 | 0.024 |
| Vm(ml/kg/min) | 5.6 | 269.8 | 91.7 | 79.8 | 107.4 | 27.6 | 20.4 | 95.5 | 25.6 | 0.199 | 0.39 |
| NIBP-SBP (mmHg) | 61 | 220 | 108 | 96 | 125 | 29 | 20.7 | 111.3 | 22.1 | 0.172 | 0.337 |
| NIBP-DBP (mmHg) | 35 | 174 | 67 | 57 | 74 | 17 | 11.8 | 66.9 | 13.8 | 0.107 | 0.211 |
| HR (/min) | 31 | 137 | 74 | 63 | 84 | 21 | 14.8 | 74.3 | 14.6 | 0.113 | 0.222 |
| SPO2(%) | 82 | 100 | 100 | 100 | 100 | 0 | 0 | 99.5 | 1.1 | 0.009 | 0.017 |
| Vigileo CO(L/min) | 1.3 | 15.3 | 4.3 | 3.6 | 5.5 | 1.9 | 1.3 | 4.6 | 1.5 | 0.012 | 0.023 |

**Table S3.** Numeric data are presented as minimum, maximum, Q-Quartile, Median, Mean, SD-Standard deviation, IQR-interquartile range, Mad-mean absolute deviation, SE-standard error, and CI-confidence interval, measured 444.1 hours of intraoperative monitoring data from 142 patients. Vigileo CO-Cardiac output measured using the Vigileo device. NIBP-SBP and NIBP-DBP- Systolic and Diastolic noninvasive blood pressure, HR-plethysmographic heart rate, FiO2-Fraction of inspired oxygen, TV- Expiratory tidal volume, Vm-expiratory minute volume, RR-respiratory rate, PEEP-positive end-expiratory pressure, PIP-peak inspiratory pressure, EtCO2- infrared spectrometry capnography, which measures end-tidal CO2.
