# Supplementary material for: A multimodal stacked ensemble model for cardiac output prediction utilizing cardiorespiratory interactions during general anesthesia
Source: Sci Rep. 2024 Mar 29;14:7478. doi: 10.1038/s41598-024-57971-6 (PMC10980739; doi:10.1038/s41598-024-57971-6)
Supplement: Supplementary file 2 — Supplementary Table S4. [file 41598_2024_57971_MOESM2_ESM.docx]

| **Best Model** | **Hyperparameters** |
| --- | --- |
| RF-NIBP/HR/MV/SpO2/EtCO2/Target CO-EV1000 | Fold_assignment="Random", ntrees=318, max_depth=40, stopping_metric="MAE", max_runtime_secs=286,  sample_rate=1, histogram_type="UniformAdaptive", categorical_encoding="Enum", calibration_method="PlattScaling", distribution="gaussian". |
| XGBoost-NIBP/HR/MV/SpO2/EtCO2/Target CO-EV1000 | Fold_assignment="Random", stopping_metric="MAE", max_runtime_secs=375, distribution="gaussian", categorical_encoding="OneHotInternal", ntrees=768, max_depth=8, learn_rate=0.09, eta=0.09, sample_rate=0.93,  subsample=0.93, col_sample_rate=0.5, colsample_bylevel=0.5, calibration_method="PlattScaling", tree_method="exact", dmatrix_type="dense", backend="cpu" |
| RF-NIBP/HR/ Target CO-EV1000 | Fold_assignment="Random", ntrees=1589, max_depth=30, stopping_metric="MAE", max_runtime_secs=361,   sample_rate=0.9, histogram_type="UniformAdaptive", categorical_encoding="Enum", calibration_method="PlattScaling", distribution="gaussian". |
| XGBoost-NIBP/HR/Target CO-EV1000 | Fold_assignment="Random", stopping_metric="MAE", max_runtime_secs=614, distribution="gaussian", categorical_encoding="OneHotInternal", ntrees=749, max_depth=9,learn_rate=0.2,eta=0.2, sample_rate=0.95,   subsample=0.95, col_sample_rate=0.76, colsample_bylevel=0.76, calibration_method="PlattScaling", tree_method="exact", dmatrix_type="dense", |
| GBM-NIBP/HR/ Target CO-EV1000 | Fold_assignment="Random", ntrees=1000, max_depth=19, min_rows=20, stopping_metric="MAE", max_runtime_secs=606, learn_rate=0.007, distribution="gaussian", sample_rate=0.4, col_sample_rate=0.7, histogram_type="UniformAdaptive", categorical_encoding="Enum", calibration_method="PlattScaling". |
| GBM-MV/SpO2/EtCO2/Target CO-EV1000 | Fold_assignment="Random", ntrees=1000, max_depth=19, stopping_metric="MAE", max_runtime_secs=512, learn_rate=0.007, distribution="gaussian", sample_rate=0.7, col_sample_rate=0.7, col_sample_rate_per_tree=0.65, histogram_type="UniformAdaptive", categorical_encoding="Enum", calibration_method="PlattScaling". |
| RF-MV/SpO2/EtCO2/Target CO-EV1000 | Fold_assignment="Random", ntrees=458, max_depth=40, stopping_metric="MAE", max_runtime_secs=272,   sample_rate=0.9, histogram_type="UniformAdaptive", categorical_encoding="Enum", calibration_method="PlattScaling", distribution="gaussian". |
| GBM-NIBP/HR/ MV/SpO2/EtCO2/Target CO-EV1000 | Fold_assignment="Random", ntrees=1000, max_depth=18, min_rows=100, stopping_metric="MAE", max_runtime_secs=399, learn_rate=0.009,distribution="gaussian", sample_rate=0.75, col_sample_rate=0.75, histogram_type="UniformAdaptive", categorical_encoding="Enum", calibration_method="PlattScaling". |
| XGBoost-MV/SpO2/EtCO2/Target CO-EV1000 | Fold_assignment="Random", stopping_metric="MAE", max_runtime_secs=614, distribution="gaussian", categorical_encoding="OneHotInternal", ntrees=582, max_depth=4, learn_rate=0.19, sample_rate=0.81,   subsample=0.81, col_sample_rate=0.56, colsample_bylevel=0.56, calibration_method="PlattScaling", tree_method="exact", dmatrix_type="dense". |
| GLM-NIBP/HR/MV/SpO2/EtCO2/Target CO-EV1000 | Fold_assignment="Random", family="gaussian", solver="IRLSM",alpha=1, lambda=0.001, max_iterations=50, objective_epsilon=1e-04, gradient_epsilon=1e-04, lambda_min_ratio=1e-04, link="identity",max_active_predictors=5000, obj_reg=2.898047e-05. |
| GLM-NIBP/HR/ Target CO-EV1000 | fold_assignment="Random", family="gaussian", solver="IRLSM",alpha=0.01, lambda=0, max_iterations=50, objective_epsilon=1e-06,  gradient_epsilon=1e-06, lambda_min_ratio=1e-04, link="identity",max_active_predictors=5000, obj_reg=2.898047e-05. |
| GLM-MV/SpO2/EtCO2/Target CO-EV1000 | family="gaussian", solver="IRLSM", alpha=0.01, lambda=0, max_iterations=50, objective_epsilon=1e-06, gradient_epsilon=1e-06, lambda_min_ratio=1e-04, link="identity", max_active_predictors=5000, obj_reg=2.898047e-05. |
| RF-NIBP/HR/MV/SpO2/EtCO2/Target CO-Vigileo | Fold_assignment="Random", ntrees=862, max_depth=50, stopping_metric="MAE", max_runtime_secs=134,  sample_rate=0.7, histogram_type="UniformAdaptive", categorical_encoding="Enum", calibration_method="PlattScaling", distribution="gaussian". |
| XGBoost-NIBP/HR/MV/SpO2/EtCO2/Target CO-Vigileo | Fold_assignment="Random", stopping_metric="MAE", max_runtime_secs=369, distribution="gaussian", categorical_encoding="OneHotInternal", ntrees=808, max_depth=9,learn_rate=0.02, eta=0.02, sample_rate=0.84,  subsample=0.84, col_sample_rate=0.82, colsample_bylevel=0.82, calibration_method="PlattScaling", tree_method="exact", dmatrix_type="dense". |
| RF-GBM-NIBP/HR/Target CO-Vigileo | Fold_assignment="Random", ntrees=2050, max_depth=50, stopping_metric="MAE", max_runtime_secs=654,  sample_rate=1, histogram_type="UniformAdaptive", categorical_encoding="Enum", calibration_method="PlattScaling", distribution="gaussian", |
| XGBoost-GBM-NIBP/HR/Target CO-Vigileo | Fold_assignment="Random", stopping_metric="MAE", max_runtime_secs=806, distribution="gaussian", categorical_encoding="OneHotInternal", ntrees=896, max_depth=9,learn_rate=0.07,eta=0.07, sample_rate=0.57,  subsample=0.57, col_sample_rate=0.84, colsample_bylevel=0.84, calibration_method="PlattScaling", tree_method="exact", dmatrix_type="dense". |
| GBM-NIBP/HR/Target CO-Vigileo | Fold_assignment="Random", ntrees=500, max_depth=16, min_rows=1, topping_metric="MAE", max_runtime_secs=591, learn_rate=0.009, distribution="gaussian", sample_rate=0.4, col_sample_rate=0.6, histogram_type="UniformAdaptive", categorical_encoding="Enum", calibration_method="PlattScaling", |
| RF-MV/SpO2/EtCO2/Target CO-Vigileo | Fold_assignment="Random", ntrees=1382, max_depth=30, stopping_metric="MAE", max_runtime_secs=219,  sample_rate=0.8, histogram_type="UniformAdaptive", categorical_encoding="Enum", calibration_method="PlattScaling",  distribution="gaussian" |
| XGBoost-NIBP/HR/Target CO-Vigileo | Fold_assignment="Random", stopping_metric="MAE", max_runtime_secs=806, categorical_encoding="OneHotInternal", ntrees=896, max_depth=9,learn_rate=0.07,eta=0.07, sample_rate=0.57, subsample=0.57, col_sample_rate=0.84, colsample_bylevel=0.84, calibration_method="PlattScaling", tree_method="exact", dmatrix_type="dense",backend="cpu", distribution="gaussian". |
| GBM-MV/SpO2/EtCO2/Target CO-Vigileo | Fold_assignment="Random", ntrees=1000, max_depth=9, min_rows=1, stopping_metric="MAE", max_runtime_secs=122, learn_rate=0.008, distribution="gaussian", sample_rate=0.85, col_sample_rate=0.8, histogram_type="UniformAdaptive",categorical_encoding="Enum", calibration_method="PlattScaling". |
| XGBoost-MV/SpO2/EtCO2/Target CO-Vigileo | Fold_assignment="Random", stopping_metric="MAE", max_runtime_secs=855, distribution="gaussian", categorical_encoding="OneHotInternal", ntrees=209, max_depth=9,learn_rate=0.18,eta=0.18, sample_rate=0.92, subsample=0.92, col_sample_rate=0.92, colsample_bylevel=0.92, calibration_method="PlattScaling", tree_method="exact", dmatrix_type="dense", |
| GLM-NIBP/HR/MV/SpO2/EtCO2/Target CO-Vigileo | Fold assignment="Random", family="gaussian", solver="IRLSM", alpha=0.01, lambda=0, max_iterations=50, objective_epsilon=1e-06, gradient_epsilon=1e-06, lambda_min_ratio=1e-04, link="identity",max_active_predictors=5000, obj_reg=8.005123e-05. |
| GLM-NIBP/HR/Target CO-Vigileo | Fold_assignment="Random", family="gaussian", solver="IRLSM",alpha=0.01, lambda=0, max_iterations=50, objective_epsilon=1e-06,gradient_epsilon=1e-06, lambda_min_ratio=1e-04, link="identity", max_active_predictors=5000, obj_reg=8.005123e-05. |
| GLM-MV/SpO2/EtCO2/Target CO-Vigileo | Fold_assignment="Random", family="gaussian", solver="IRLSM",alpha=0.01, lambda=0, max_iterations=50, gradient_epsilon=1e-04, link="identity", lambda_min_ratio=1e-04, max_active_predictors=5000, obj_reg=8.005123e-05. |

**Table S4.** List of hyperparameter values for best base models.
