# Supplementary material for: A multimodal stacked ensemble model for cardiac output prediction utilizing cardiorespiratory interactions during general anesthesia
Source: Sci Rep. 2024 Mar 29;14:7478. doi: 10.1038/s41598-024-57971-6 (PMC10980739; doi:10.1038/s41598-024-57971-6)
Supplement: Supplementary file 3 — Supplementary Table S5. [file 41598_2024_57971_MOESM3_ESM.docx]

|  | **EV1000 (n=327)** | **Vigileo (n=142)** |
| --- | --- | --- |
| Sevoflurane | n=44 | n=23 |
| Inspired sevoflurane (kPa) | 1.83± 0.67 | 1.71±0.69 |
| Expired sevoflurane (kPa) | 1.64±0.52 | 1.55± 0.52 |
| MAC sevoflurane | 0.80±0.27 | 0.83±0.32 |
| BIS | 43.69±10.97 | 41.02±9.90 |
| Desflurane | n=232 | n=40 |
| Inspired desflurane (kPa) | 5.55±1.52 | 6.21±1.24 |
| Expired desflurane (kPa) | 5.09±1.35 | 5.82±1.11 |
| MAC desflurane | 0.88±0.23 | 0.98±0.17 |
| BIS | 40.70±9.46 | 38.68±8.21 |
| Propofol | n=51 | n=79 |
| Propofol consumption rate (mg kg−1·minute−1) | 0.11±0.04 | 0.13±0.07 |
| BIS | 40.68±7.64 | 41.67±7.82 |

**Table S5.** Sevoflurane and Desflurane were administered via an anesthesia machine (Primus, Dräger, Lübeck, Germany). Propofol was delivered using a target-controlled infusion pump (Orchestra Base Primea with module DPS; Fresenius Kabi AG, Bad Homburg, Germany). An EEG-based depth anesthesia monitoring system, utilizing a brain monitor (BIS VistaTM, Medtronic, Dublin, Ireland), was employed to assess the depth of anesthesia.
